# Supplementary material for: Direct Photochemical Synthesis of Substituted Benzo[b]fluorenes
Source: Org Lett. 2024 Nov 25;26(48):10364–8. doi: 10.1021/acs.orglett.4c03978 (PMC11629382; doi:10.1021/acs.orglett.4c03978)
Supplement: Supplementary file 2 — ol4c03978_si_002.zip [file ol4c03978_si_002.zip › FID for Publication/20240606_RC-2-C-4-90A_01/plots/RC-2-C-4-90A_CARBON_20240606_01_plot01.pdf]

RC-2-C-4-90A

Location **27**
Date collected **2024-06-06**
Pulse sequence **CARBON**
Solvent **cdcl3**
Temperature **25**
Spectrometer **localhost.localdomain-vnmrs500**
Suboperator **RuairiCrawford**
Operator **MBaumann**

SAMPLE

date	Jun 6 2024
solvent	cdcl3
file	/home/data/MBaumann/cscb500/RuairiCrawford/2024/20240606_RC-2-C-4-90A_01/RC-2-C-4-90A_CARBON_20240606_01.fid

ACQUISITION

sw	31250.0
at	1.049
np	65536
fb	17000
bs	8
ss	128
d1	1.000
nt	2000
ct	2000

TRANSMITTER

tn	C13
sfrq	125.668
tof	1913.4
tpwr	58
pw	7.700

DECOUPLER

dn	H1
dof	0
dm	yyy
decwave	w
dpwr	36
dmf	13423

PRESATURATION

satmode	n
wet	n

SPECIAL

temp	25.0
gain	50
spin	0
hst	0.008
pw90	15.400
alfa	10.000

FLAGS

il	n
in	n
dp	y
hs	nn

PROCESSING

lb	0.50
fn	not used

DISPLAY

sp	-1803.0
wp	31250.0
rfl	1803.0
rfp	0
rp	-122.8
lp	0

PLOT

wc	169
sc	8
vs	5218
th	3
ai cdc ph	

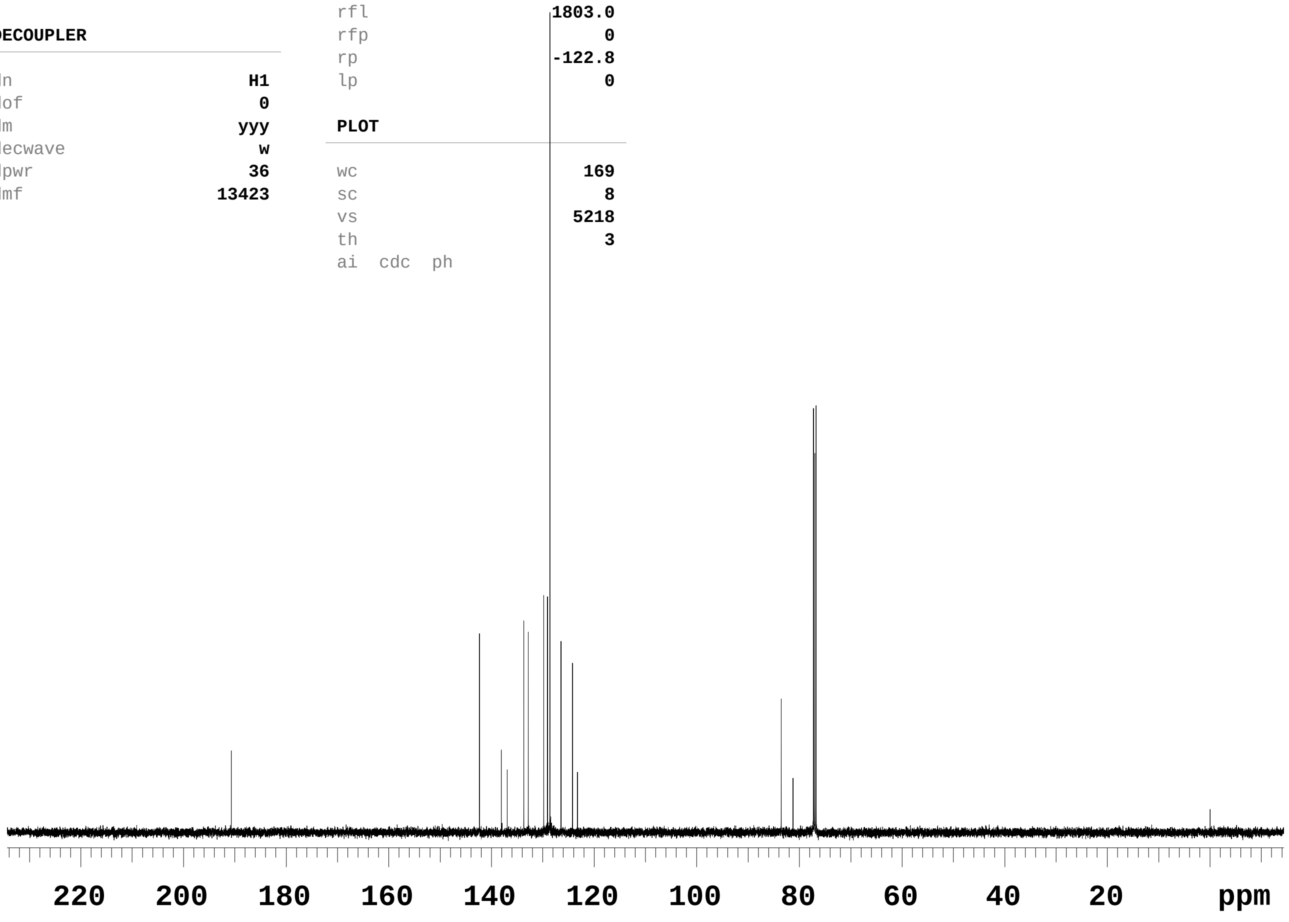

Plotname: RC-2-C-4-90A_CARBON_20240606_01_plot01

Data file /home/data/MBaumann/cscb500/RuairiCrawford/2024/20240606_RC-2-C-4-90A_01/RC-2-C-4-90A_CARBON_20240606_01.fid
Plot date 2024-06-06
